# EF-Net: Mental State Recognition by Analyzing Multimodal EEG-fNIRS via CNN

**DOI:** 10.3390/s24061889

**Published:** 2024-03-15

**Authors:** Aniqa Arif, Yihe Wang, Rui Yin, Xiang Zhang, Ahmed Helmy

**Affiliations:** 1 Department of Computer Science, University of North Carolina, Charlotte, NC 28223, USAahmed.helmy@charlotte.edu (A.H.); 2 Department of Health Outcomes and Biomedical Informatics, University of Florida, Gainesville, FL 32611, USA

**Keywords:** deep learning, multimodal, EEG, fNIRS, brain activity learning

## Abstract

Analysis of brain signals is essential to the study of mental states and various neurological conditions. The two most prevalent noninvasive signals for measuring brain activities are electroencephalography (EEG) and functional near-infrared spectroscopy (fNIRS). EEG, characterized by its higher sampling frequency, captures more temporal features, while fNIRS, with a greater number of channels, provides richer spatial information. Although a few previous studies have explored the use of multimodal deep-learning models to analyze brain activity for both EEG and fNIRS, *subject-independent* training–testing split analysis remains underexplored. The results of the subject-independent setting directly show the model’s ability on unseen subjects, which is crucial for real-world applications. In this paper, we introduce *EF-Net*, a new CNN-based multimodal deep-learning model. We evaluate EF-Net on an EEG-fNIRS word generation (WG) dataset on the mental state recognition task, primarily focusing on the subject-independent setting. For completeness, we report results in the subject-dependent and subject-semidependent settings as well. We compare our model with five baseline approaches, including three traditional machine learning methods and two deep learning methods. EF-Net demonstrates superior performance in both accuracy and F1 score, surpassing these baselines. Our model achieves F1 scores of 99.36%, 98.31%, and 65.05% in the subject-dependent, subject-semidependent, and subject-independent settings, respectively, surpassing the best baseline F1 scores by 1.83%, 4.34%, and 2.13% These results highlight EF-Net’s capability to effectively learn and interpret mental states and brain activity across different and unseen subjects.

## 1. Introduction

Recognizing mental states by measuring brain activity has long been a critical challenge in neuroscience. Its complexity arises from the intricate nature of the brain area, the necessity of employing noninvasive methods when measuring brain signals, and the requirement of maintaining consciousness and comfort of the individual for accurate brain activity assessment. Electroencephalography (EEG) has been extensively studied as a standard method, allowing neuronal electrical signals to be conveniently measured through electrodes placed on the scalp [1,2,3]. Another significant brain response, the blood oxygen level-dependent (BOLD) signal, is derived from the brain’s blood oxygen levels, and is coupled with the brain’s underlying electrical activity. Functional near-infrared spectroscopy (fNIRS) has emerged as a low-cost method for measuring the BOLD response, replacing the expensive and intricate acquisition of functional magnetic resonance imaging (fMRI) data. fNIRS records changes in optical density through the skull to determine the blood oxygen levels in different brain regions [4,5,6]. A notable feature of fNIRS is its compatibility with EEG, allowing for simultaneous data recording using electrodes placed on the head. The availability of skullcaps equipped with both NIRS and EEG electrodes facilitates this simultaneous measurement, enabling the analysis of data from both modalities on the same subject and functional task [7,8].

In recent years, the advent of modern deep learning techniques has significantly impacted the field of brain analysis [9,10,11]. Unlike traditional machine learning methods, which rely heavily on manual feature extraction, deep learning approaches can analyze EEG and fNIRS data multimodally without requiring exhaustive preliminary processing. This advancement is particularly beneficial in handling the coherent data generated by these two modalities, allowing for more efficient and comprehensive analysis [12]. EEG is known for its low spatial and high temporal resolutions, providing near-immediate measurement of spikes related to brain activity. Conversely, while fNIRS offers high spatial resolution, it has limited temporal resolution owing to the delay in detecting changes in blood oxygen levels. Employing a multimodal deep learning model allows for the extraction of spatial and temporal features, leveraging the complementary strengths of EEG and fNIRS in brain activity analysis.

However, existing methods in the field of brain activity learning have predominantly focused on subject-dependent and subject-semidependent analysis [12,13,14], where samples from the same subject can appear in both the training and test sets. While these approaches are effective in certain scenarios, such as creating a personalized model, they place significant limits on broader applicability to unseen subjects. Consequently, there is an essential need for methodologies that are capable of generalizing across a diverse range of subjects, including those not encountered during the model’s training phase. Implementing such methodologies would substantially enhance the universality and practicality of brain activity analysis, contributing to a more comprehensive understanding of neural patterns and behaviors. However, due to the low information-to-noise ratio, achieving generalization to unseen subjects is quite challenging. Each subject has unique characteristics and patterns that may obscure the acquisition of general features across subjects, and the distribution of data for each subject can be markedly different even for those with the same label [15,16].

These limitations and challenges motivate us to explore the effectiveness of the multimodal model in cross-subject brain activity learning. In this paper, we introduce *EF-Net*, a convolutional neural network (CNN)-based deep learning method tailored to multimodal cross-subject analysis of mental state recognition via EEG and fNIRS data. Our approach includes analyses in various settings: subject-dependent, subject-semidependent, and subject-independent. This multifaceted strategy allows for a more flexible and versatile application of the models, empowering them to learn and adapt to a broader spectrum of neural data and mental states irrespective of the subjects’ previous interactions with the model [15]. We evaluated our EF-Net model on the EEG-fNIRS WG dataset from [17], achieving impressive F1 scores of 99.36% in subject-dependent analyses, 98.31% in subject-semidependent analyses, and 65.05% in subject-independent analyses. In the respective training–testing splits, EF-Net surpassed the baseline models by margins of 1.83%, 4.34%, and 2.13% when using both EEG and fNIRS data. Furthermore, we observed that the combined use of EEG and fNIRS data yields superior results, particularly when using the subject-independent setting. This finding underscores EF-Net’s effectiveness in generalizing to unseen subjects, demonstrating its potential applicability in real-world scenarios.

In summary, our paper’s key contributions are as follows:

- We introduce a CNN-based deep-learning method named *EF-Net* for fNIRS-EEG multimodal cross-subject analysis in brain activity representation learning, and apply our method to a mental state recognition task.
- We explore the challenges associated with learning a general model for cross-subject brain activity analysis, then conduct detailed experiments across various settings, including the employment of single or multiple modalities and different subject-dependent, subject-semidependent, and subject-independent training–testing splits.
- The effectiveness of *EF-Net* is empirically demonstrated, showcasing its potential in leveraging multiple modalities in order to generalize to unseen subjects through representation learning.

The rest of this document is organized as follows. Section 2 provides an overview of the current literature and the state of research in this field. Section 3 describes the preprocessing of the dataset, followed by an in-depth description of the proposed model architecture. In Section 4, we present our experiments conducted with EF-Net and all baselines; this section additionally covers the training and testing setups and reports the obtained results. Finally, Section 7 discusses our findings and concludes the paper.

## 2. Related Work

Brain-computer interfaces (BCI) have revolutionized our interactions with technology, creating direct links between the brain and electronic devices [18]. Incorporating various noninvasive methods such as EEG [19,20], fNIRS [21], eye-tracking [22,23], and VR/AR integrations [24,25], BCIs promise wide-ranging applications. These include facilitating communication for those with disabilities [19,26] and enriching immersive gaming and virtual reality experiences [27], as well as roles in disease diagnosis [28,29] and mental state monitoring [30,31]. BCI technology’s expanding abilities herald a new frontier in healthcare, entertainment, and education, marking a significant leap in human–computer interaction.

Existing work in the hybrid EEG-fNIRS domain is relatively sparse, with experiments varying significantly in quality and methodology. Most studies to date have employed traditional machine learning methods, with linear discriminant analysis (LDA) and support vector machine (SVM) classifiers being particularly prominent. Only a few studies have reported subject-semidependent results [17,32]. Many of these studies have instead relied on subject-dependent models, where the mean accuracy is reported as an average of individual subjects’ accuracies, as seen with LDA in [33,34], k-nearest neighbors (KNN) in [35], and SVM in [13,36,37]. The mean accuracy for subject-dependent hybrid tests has reached as high as 91.02% in certain studies [34,36]. However, a significant limitation of traditional ML methods is the extensive feature extraction process that they require, with additional techniques such as principal component analysis (PCA), channel selection, spectral features, and wavelet transforms being used as well.

Deep learning applied to multimodal EEG-fNIRS data has only been explored in a small number of studies. The study by [12] employed a straightforward fully connected network trained on EEG-fNIRS data for motor imagery tasks in a subject-semidependent setting. The M2NN study [38] showcased feature extraction using a custom-branched CNN, testing it on both EEG and fNIRS modalities for motor imagery tasks within a leave-one-out subject-independent setting. The research in [39] introduced an EEG-fNIRS hybrid brain–computer interface (BCI). This study applied CNNs to classify overt and imagined speech, first creating two separate subnets for each modality, then fusing and processing them through gated recurrent units (GRUs). FGANet [14] implements an fNIRS-guided attention feature in a CNN along with EEG and fNIRS convolution branches to form a fusion network for classifying mental arithmetic and imagery tasks in a subject-semi-dependent setting. A study by [40] explored the use of a deep recurrent neural network (RNN) for seizure detection with EEG-fNIRS data, again in a subject-semidependent setting. The research in [17] employed an LSTM to classify mental workload using n-back tests, with a focus on subject-semidependent analysis.

Existing works reveal a notable gap in the multimodal deep learning literature, particularly around studies conducting extensive testing comparisons across subject-dependent, subject-semidependent, and subject-independent setups, as our paper does. For the differences between these three training–testing split settings, see Section 4.3 and Section 5. Additionally, comprehensive comparisons between traditional machine learning and deep learning methods are scarce. Below, we outline our deep learning approach and the results from our systematic comparison of various machine learning techniques.

## 3. Dataset and Method

This section details the dataset integrating EEG and fNIRS signals, the preprocessing specifics, and the EF-Net method introduced in this study.

### 3.1. Dataset: EEG-fNIRS

We aimed to find a dataset satisfying the following requirements: (a) a focus on mental state recognition tasks, (b) a substantial number of subjects to ensure generalizability, and (c) the inclusion of data acquired through multiple modalities. Only a few open-access EEG and fNIRS datasets satisfied all of these requirements. The EEG-fNIRS WG dataset is an open-access resource comprising simultaneous recordings of both EEG and fNIRS signals, released by [17], and is one of only two open-access datasets available for EEG and fNIRS hybrid data. This dataset is divided into three sub-datasets, A, B, and C, each corresponding to distinct tasks. We utilized Dataset C for our study of mental state recognition, as it includes many subjects and is suitable for classification tasks.

The dataset contains 26 subjects, with each subject participating in 60 trials spread across three sessions. In each session, 20 trials were conducted in random order, comprising 10 Word Generation (WG) and 10 Baseline (BL) trials. The dataset is therefore balanced between the two classes. The fNIRS data were captured using 72 channels at a sampling rate of 10 Hz, while the EEG data were recorded from 30 channels at 1000 Hz. The fNIRS and EEG electrodes were mounted on the same cap, facilitating simultaneous data acquisition. Each trial was randomly assigned as either a WG trial or a BL trial. In a WG trial, participants were shown a single letter for 2 s, followed by a blank screen for 10 s, during which period they were instructed to think of new words starting with a given letter. For the BL trial, a fixation cross was displayed instead of a blank screen; participants were asked to relax and focus on the cross in order to establish a baseline or resting cognitive load. Both trial types concluded with a rest period of 13–15 s featuring the fixation cross.

### 3.2. Data Preprocessing

The dataset is provided as MATLAB struct files, which can be processed in Python using the SciPy Library. The result includes EEG and fNIRS signal data along with timestamps marking the start of each trial. The fNIRS data comprise two arrays, one for deoxygenated data from 36 channels and another for oxygenated data from 36 channels, resulting in 72-channel data. Basic data preprocessing was already performed by the original publishers [17]. For the fNIRS signals, the optical intensity measurements from the electrodes were converted into HbO (oxygenated) and HbR (deoxygenated) oxygen concentration values and then downsampled to 10 Hz. The EEG signals were processed by downsampling the raw electrode data to 200 Hz and applying a bandpass filter from 1–40 Hz. In addition, ocular artifacts were removed from the EEG signals using the EEGLAB toolbox.

Further preprocessing steps were based on our preliminary tests. We discarded the initial 2 s of instruction time, retained the subsequent 10 s of task implementation, and excluded the rest of the period data. This approach yielded a consistent time window of 10 s per trial for analysis. The initial EEG sampling frequency of 200 Hz led to an EEG matrix of size 2000 × 30 (time samples × channels), while the fNIRS sampling frequency of 10 Hz resulted in an NIRS matrix of size 100 × 72 (time samples × combined deoxy and oxy channels). We then applied additional downsampling, reducing the amount of data by half, resulting in an EEG matrix of 1000 × 30 with a sampling frequency of 100 Hz and an fNIRS matrix of 50 × 72 with a sampling frequency of 5 Hz. After segregating the tasks, we utilized a sliding window of 5 s with an overlap of 1 s for each window. This process produced six task samples from each trial, amounting to 360 task samples per subject, with each sample labeled WG or BL according to the original trial label. Consequently, we obtained 9360 5 s task samples with dimensions of 500 × 30 (time samples × channels) for EEG and 25 × 72 (time samples × combined deoxy and oxy channels) for fNIRS. The data samples were normalized along channels using Z-Score normalization from the StandardScaler library in SciKit Learn [41]. The information of the processed dataset is provided in Table 1.

### 3.3. The EF-Net Method

**Figure 1:**
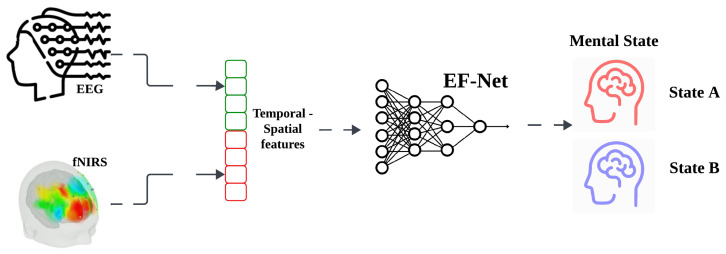
**Overview of EF-Net.** Our EF-Net takes EEG and fNIRS data as input to extract temporal and spatial features simultaneously for mental state classification.

This paper introduces EF-Net, a multimodal deep learning model for mental state classification utilizing the capabilities of EEG to extract temporal features and fNIRS to extract. EF-Net comprises two specialized branches, each dedicated to processing one of these modalities. The overview of the EF-Net pipeline is illustrated in Figure 1. The EEG branch is specifically designed to focus on extracting temporal features, and takes an EEG signal $X_{e}\in R^{T_{e}\times C_{e}}$ as input, where $T_{e}=500$ represents the number of time stamps and $C_{e}=30$ denotes the number of channels. Conversely, the fNIRS branch aims to capture spatial features, processing an fNIRS signal $X_{f}\in R^{T_{f}\times C_{f}}$, where $T_{f}=25$ and $C_{f}=72$ represent the timestamps and channel numbers, respectively. Both branches of EF-Net independently learn data representations through a sophisticated deep learning architecture. Subsequently, these representations are concatenated and passed through fully connected networks, leading to the final classification output. In practice, the EF-Net takes EEG and fNIRS data minibatches as input. We built EF-Net using Tensorflow 2.0 Keras API [42] and ran all of our experiments on Google Colab using a V100 GPU.

#### 3.3.1. EEG Modality Branch

The input EEG samples, initially shaped as (500 × 30), are transformed into (500 × 30 × 1) to facilitate compatibility with 2D convolution. The effectiveness of applying 2D convolution to 1D temporal data has been validated in numerous studies, such as [10,20]. EF-Net employs three Conv2D blocks, each with a kernel size of (7 × 1) and 32 filters, to process these input samples. The chosen kernel size (7 × 1) was specifically selected to scan temporal features along timestamps across a single channel. The repetitive Conv2D blocks are intended to capture a broad range of features, from low-level to high-level. Following this, a max pooling layer with a pooling size of (7 × 1) is applied, retaining the most prominent features from the convolutional feature matrices. These layers are subsequently accompanied by a dropout layer with a rate of 0.5, effectively turning certain perceptrons off within that part of the model to prevent overfitting [43]. Batch normalization [44] is applied after the dropout layer to mitigate distribution shifts among batch samples and accelerate the training process.

After these layers, three more Conv2D blocks are introduced, each with a kernel size of (4 × 4) and 64 filters. This kernel configuration (4 × 4) is tailored to scan features across four channels along the timestamps, allowing both temporal and spatial features to be captured. A max pooling layer with a pooling size of (4 × 4) follows, effectively reducing dimensions in both temporal and spatial domains. Dropout and batch normalization are incorporated at this stage. The final output is then flattened into a representation of 4480 elements. Two fully connected layers with hidden dimensions of 256 and output dimensions of 128 are utilized to produce the final output of the EEG modal.

#### 3.3.2. fNIRS Modality Branch

In a similar manner to the EEG branch, the fNIRS samples, originally shaped as (25 × 72), are adapted to fit 2D convolution by reshaping them to (25 × 72 × 1). Due to the significantly lower number of timestamps in the fNIRS samples compared to the EEG samples, only two Conv2D blocks are employed for this branch. Each block has a kernel size of (4 × 1) and 32 filters, specifically chosen to extract temporal features from the fNIRS data. Following these convolutional blocks, a series of layers—max pooling, dropout, and batch normalization—are applied to refine the feature extraction process.

Subsequently, two additional Conv2D blocks are introduced, each with a kernel size of (2 × 2) and 64 filters, with the aim of extracting both temporal and spatial features from the fNIRS samples. These blocks are again followed by max pooling, dropout, and batch normalization to enhance the model’s performance and generalization capabilities. After processing through these layers, the output is flattened into a representation and fed into a single fully connected network layer with an output dimension of 128. This step produces the final representation for the fNIRS model.

#### 3.3.3. Combining the Two Branches

Generally, EEG samples have more timestamps, capturing more temporal features, while fNIRS samples, with their greater number of channels, contain more spatial features. Thus, we have two distinct representations, each with a length of 128, corresponding to these two modalities. These are concatenated to form a unified representation of 256 elements, effectively merging the compressed temporal and spatial information. This combined representation is fed into a fully connected layer with an output dimension of 256. To prevent overfitting, dropout and L2 regularization are applied following this layer. Subsequently, an additional layer with an output dimension of 64 is added. Finally, the model outputs the probability of the binary classification labels using a sigmoid activation function. Except for the last layer, all activation functions in the EF-Net model, including both branches, are set to ReLU [45]. For our experiments involving single-modality input, we deactivated one branch of our complete EF-Net model and fed only the single-modality data into the active branch. The fully connected network remained the same as in the original model’s configuration.

EF-Net has a relatively manageable number of parameters, totaling 1,757,314 trainable parameters, significantly fewer than the 30–100 million parameters typically found in other popular deep network architectures [46,47,48]. This reduction in parameters leads to a marked improvement in training performance, especially when contrasted with baseline deep learning approaches.

## 4. Experiments

We compared our method with five baseline approaches, including both traditional machine learning (ML) and deep learning (DL) techniques. For the ML baselines, we selected three widely used algorithms: Support Vector Machine classification (SVM), Random Forest (RF), and K-nearest neighbors (KNN). In terms of DL baselines, we chose two popular benchmark methods: Visual Geometry Group—Very Deep Convolutional Networks (VGG) [47] and Residual Network—50 Layers (Resnet50) [46].

All experiments were conducted using three random seeds, which were employed in order to shuffle either the subjects (in the case of subject-independent settings) or the samples themselves (in all other settings). We used seeds of 38, 43, and 45, and report the average results across these three seeds. The results of each experiment are presented with scores that include Accuracy, Precision, Recall, F1, and/or ROC-AUC. All scores represent the model’s performance on the testing set. Our evaluation encompassed three distinct data usage settings: only the fNIRS dataset, only the EEG dataset, and a combination of both. Additionally, we conducted experiments in subject-dependent, subject-semidependent, and subject-independent training–testing split settings. This comprehensive approach resulted in nine distinct settings (3 × 3) for evaluation.

**Figure 2:**
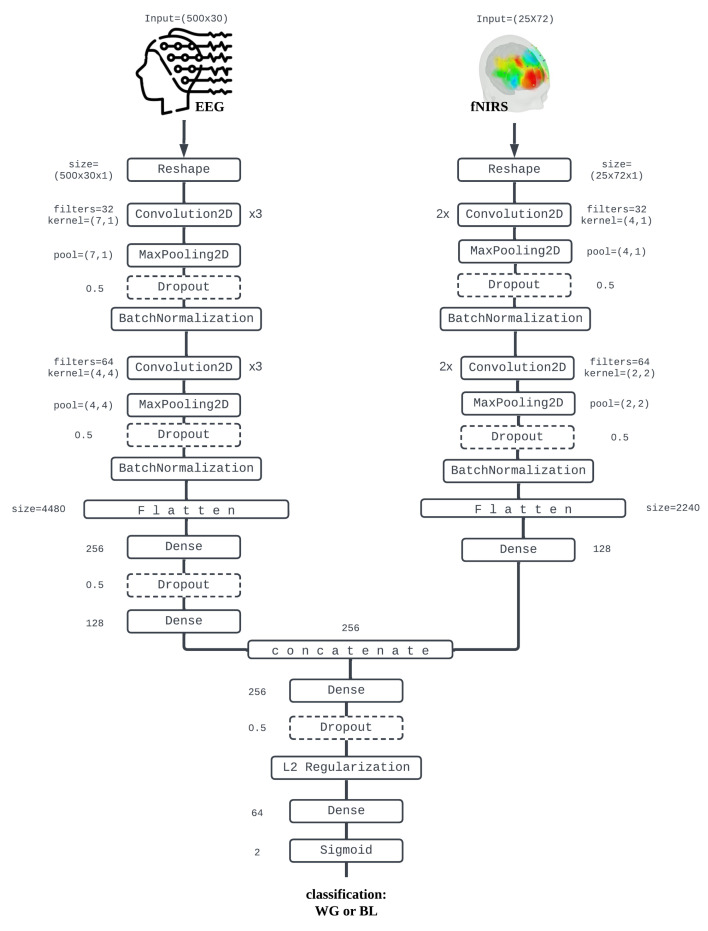
**Details of EF-Net structure.** This figure illustrates the intricate structure of our EF-Net model, which is composed of two branches tailored to process two distinct modalities: EEG and fNIRS. To facilitate compatibility with the Conv2D kernel, the input data for both EEG and fNIRS are reshaped to include an additional dimension. Conv2D blocks are employed to extract temporal and spatial features effectively, akin to the methods described in [10,20]. The annotations ×3 and ×2 indicate that certain blocks are “repeated three times” and “repeated two times”, respectively. To mitigate overfitting and address sample distribution shifts during training, we incorporate max pooling, dropout, and batch normalization into the model. Due to the larger number of timestamps in EEG data, more Conv2D blocks are utilized in this branch. The learned representations from both modalities are then flattened and merged into a single comprehensive representation. L2 regularization is applied to further reduce the risk of overfitting. Finally, fully connected networks and a sigmoid layer are used to produce the mental state classification output.

The implementation details of our model are illustrated in Figure 2 and discussed in Section 3.3. The parameter tuning for our method and the deep learning baselines are included in Table A1 of Appendix A. For the ML and DL baselines, the implementation specifics are covered in Section 4.1 for the ML baselines and Section 4.2 for the DL baselines. The training–testing split settings are elaborated in Section 4.3. Section 4.4, Section 4.5 and Section 4.6 analyze the importance of different data split settings and report the corresponding results.

### 4.1. Machine Learning Baselines

The input shape of the EEG samples is (500, 30) and that of the fNIRS samples is (25, 72). To ensure compatibility with the ML baselines, we flattened our 2D samples into 1D vectors. This process yielded length vectors of 15,000 for the EEG samples and 1800 for the fNIRS samples. For the multimodal input, we combined fNIRS and EEG data and concatenated these two vectors, resulting in a combined input vector of length 16,800. We utilized SciKit Learn [41] to implement SVM, RF, and KNN, operating with default parameters. We performed some parameter tuning for the three machine learning baselines with EEG-fNIRS modalities. The results are reported in Table A2, Table A3 and Table A4 of Appendix A. Overall, these baselines did not outperform the deep learning baselines, which is intuitive.

### 4.2. Deep Learning Baselines

In addition to traditional ML models, we conducted experiments with deep learning models, specifically VGG and ResNet. Deep learning methods are typically optimized for datasets with a large number of samples. Considering that VGG and ResNet are primarily designed for image representation learning and expect 3D input matrices (height, width, channels), our 2D EEG and fNIRS datasets (timestamps, channels) required adaptation. For the DL baseline methods, we transformed our inputs into a three-channel format as follows. First, each 5 s window of samples was segmented into the three channels, with some repetition involved. For EEG, this involved 500 timestamps split into three channels as 0–200, 150–350, and 300–500, while for fNIRS it involved a simple repeat of all timestamps as 0–25, 0–25, and 0–25. This process resulted in each input sample being reshaped into a three-dimensional form, enabling the use of the VGG and ResNet models with our data.

Our approach includes two branches for the two modalities: EEG and fNIRS. Due to the size of the ResNet50 model, we used it for train with the EEG data and employed VGG for the fNIRS branch in one baseline configuration. In another baseline setup, we simply used VGG for both the EEG and fNIRS branches. Thus, our two baseline configurations were ResNet50 (EEG) + VGG16 (fNIRS) and VGG19 (EEG) + VGG16 (fNIRS). The last layer of each branch was flattened and concatenated to form a unified representation vector. We then introduced a fully connected network that utilized this representation for the final classification task.

In the single-modality experiments, we deactivated one branch of the full model and only input the data from a single modality into the remaining active branch. Specifically, for the EEG-only experiments, we utilized one baseline incorporating a VGG19 branch and another employing a Resnet50 branch, while for the fNIRS-only experiments we conducted one baseline with a VGG16 branch and another with a Resnet50 branch. All single-modality experiments features were the same as those in the original model.

These two baselines were implemented using the Tensorflow 2.0 Keras API. The learning rates were adjusted based on the rate of change in model accuracy. Batch sizes were generally maintained at 64 samples per batch, although some experiments used 32 samples per batch. The Adam optimizer was utilized to optimize model learning, with a binary cross-entropy loss function guiding the model. The highest testing accuracy and corresponding model weights from each experiment were retained.

### 4.3. Training and Testing Settings

We evaluated five baseline models and our EF-Net across three different training–testing split settings: subject-dependent, subject-semidependent, and subject-independent. The results for each setting are presented in Table 2, Table 3, and Table 4, respectively.

1. Subject-Dependent: In this setting, both training and testing are performed using different parts of samples from the same individual subject.
2. Subject-Semidependent: Here, the training and testing sets may include samples from any subject. We shuffled all samples from all subjects together before splitting them into training and testing sets.
3. Subject-Independent: This approach involves using certain subjects exclusively for training and others exclusively for testing, ensuring no overlap of samples from any specific subject between the training and testing splits. With an 80–20 split, approximately twenty subjects were utilized for training, while a separate set of six subjects were reserved for testing.

### 4.4. Subject-Dependent Results

The subject-dependent setting ensures that the model effectively learns the unique brain activity patterns of a specific individual. This capability allows the model to accurately classify any new data received from the same subject. Such an approach is particularly relevant in Brain–Computer Interface (BCI) applications, where patient-specific calibrations can assist the model in fine-tuning its response to that particular subject, leading to more accurate responses to future BCI commands.

**Setup**. We conducted experiments individually on subjects 1, 2, and 3 from our dataset. For each subject, we applied all baseline models using three random seeds and report the average results. In each experiment, the data from each single subject are divided into two parts, with 80% allocated for training and the remaining 20% used for testing.

**Results**. The average results for subjects 1, 2, and 3 are presented in Table 2. EF-Net demonstrates success in 10 out of 15 tests across the three data usage scenarios. Notably, EF-Net’s F1 score performance surpasses that of the best baseline, VGG16 and VGG16 + VGG19, by 7% on the fNIRS data and 1.83% on the combined fNIRS and EEG data. It can be observed that EF-Net yields excellent results in the subject-dependent setting when using only fNIRS data, achieving the best performance across all baselines and data settings. We speculate that the reason for this might be that the subject-dependent setting involves a smaller number of samples, where utilizing multimodal approaches could lead to overfitting.

### 4.5. Subject-Semiependent Results

Subject-semidependent experiments are designed to test models’ generalization capabilities across all subjects, thereby ensuring that the overall model performs effectively and consistently among different individuals.

**Setup**. We extracted all samples from subjects 1 to 26 and shuffled the entire dataset. The samples were split into two subsets, with 80% used for training and 20% for testing.

**Results**. The outcomes of the subject-semidependent experiment are detailed in Table 3. EF-Net outperforms the others in 12 out of 15 tests across the three data usage scenarios in this setting. Specifically, EF-Net’s F1 score outperforms the best baselines, VGG16 and VGG16 + VGG19, by margins of 2.63% for the fNIRS data and 4.34% for the combined fNIRS and EEG data modalities. Consistent with our previous findings, utilizing fNIRS data exclusively yields the most favorable results, surpassing all other baselines and settings.

### 4.6. Subject-Independent Results

The subject-independent setting, especially in the context of evaluating unseen patients or subjects, is crucial for developing a diagnostic or medical aid that is both resilient and universally applicable [15]. Each subject exhibits unique characteristics and cognitive activities, based on which certain consistent brain activity patterns may be identified and generalized. In order to effectively learn these patterns in a generic way, it is essential to conduct extensive training across a diverse range of subjects. Moreover, the model structure needs to be able to recognize features in any unseen subject, ensuring its applicability across different individuals.

**Setup**. After shuffling all the subjects using consistent random seeds, we partitioned the dataset into two groups, with 80% (twenty subjects) used to training the model and the remaining 20% (six subjects) used for testing. After partitioning the subjects, we gathered each of their respective samples for training and evaluation.

**Results**. The results for the subject-independent experiments are presented in Table 4. EF-Net outperforms the others on 11 out of 15 tests across all three data usage scenarios in this setting. Specifically, EF-Net’s F1 score exceeds the best baseline models, VGG16 and VGG16 + VGG19, by margins of 2.48% for the fNIRS data and 2.13% for the combined fNIRS and EEG data modalities. EF-Net demonstrates robust multimodal learning capability in the subject-independent setting, particularly when utilizing both EEG and fNIRS data. This outcome substantiates the hypothesis that integrating the EEG and fNIRS modalities can enhance the model’s ability to learn cross-subject features and generalize to unseen subjects, making it highly applicable to real-world scenarios.

## 5. Discussion

The challenges in subject-independent settings come from the unique noisy characteristics of each subject. Even when sharing the same class label, subjects might exhibit different data distributions [15,16]. The higher accuracy and F1 score observed in the subject-dependent and subject-semidependent settings can be attributed to potential “information leakage”, where the model learns specific subjects’ distributions during training. Consequently, these settings are best suited for training personalized models or verifying dataset effectiveness, while their real-world applicability remains somewhat limited. In contrast, the difficulty in subject-independent settings arises from the need to perform effectively on unseen subjects with potentially varying distributions. The noisy characteristics unique to each subject can obscure the generalizable features across subjects. The objective is then to learn overarching features while discarding subject-specific noise.

Recent work on this topic, such as the M2NN method proposed by [38], shares a similar objective with ours, namely, to automatically extract features using deep learning. They utilized another open-access dataset made available by [33] for motor imagery classification, which includes a comparable number of subjects to the dataset used in our study. Their model employs convolutional blocks to extract features from each modality, subsequently combining them to make a class prediction. The primary distinction lies in their use of significantly smaller and one-dimensional kernels, whereas our approach incorporates both one-dimensional temporal kernels and two-dimensional spatial kernels.

In addition, there are other types of brain–computer interface (BCI) applications beyond EEG-fNIRS that could be integrated with EEG-fNIRS for various engineering purposes. For instance, combining EEG-fNIRS data with eye tracking could substantially enhance the capabilities of VR devices. A VR helmet could, for example, be engineered to simultaneously gather these types of data. Eye tracking data could then be employed to trace the user’s gaze, allowing the virtual environment to dynamically adapt and align with the user’s line of sight. Furthermore, EEG-fNIRS data could unlock a wide range of potential applications within VR devices. For instance, when integrated with VR, EEG-fNIRS technology could offer novel approaches for managing stress, anxiety, and other mental health issues by facilitating therapeutic scenarios that adjust to the user’s mental state in real time. Moreover, this technology could provide adaptive interfaces or control mechanisms specifically designed for users with disabilities, thereby improving the accessibility and enjoyment of VR by modifying the experience to suit the user’s cognitive and emotional states.

## 6. Limitations and Future Work

Our work successfully demonstrates how combining multiple modalities can improve both accuracy and F1 score in subject-independent settings compared to single-modality approaches. We believe that this finding will benefit future research in this field. One limitation of our method is that the results in the subject-independent setting require improvement. Moving forward, we plan to extend EF-Net’s application to a wider range of EEG-fNIRS multimodal brain activity learning tasks beyond mental state recognition. Additionally, we aim to collect new datasets and explore state-of-the-art model architectures such as transformer-based models for multimodal brain activity learning [48,49,50]. In particular, we will try to explore any methods that can improve the subject-independent results, such as removing outliers in the dataset.

## 7. Conclusions

This paper introduces EF-Net, a convolutional neural network (CNN)-based multimodal learning framework designed for the analysis of brain signals in mental state recognition tasks. Leveraging both EEG and fNIRS data, EF-Net is engineered to capture the temporal and spatial features inherent in these modalities. We conducted experiments under various settings, exploring the utilization of EEG alone, fNIRS alone, and their combination. This study evaluates different training–testing split scenarios, including subject-dependent, subject-semidependent, and subject-independent configurations. We report promising results in both the subject-dependent and subject-semidependent settings, underscoring EF-Net’s effectiveness in facilitating personalized models. Although the performance in the subject-independent setting is modest and highlights areas for future improvement, our findings affirm the benefits of integrating the EEG and fNIRS modalities. This integration notably enhances the model’s ability to learn and apply cross-subject representations to unseen subjects. These results underscore EF-Net’s potential for real-world applications in mental state recognition using brain signals, and pave the way for future research on combining multiple modalities for brain activity learning.

## Figures and Tables

**Table 1:** **Processed Dataset.** Information of the processed dataset. The # stands for the number.

Data	# of Subjects	# of Samples	Timestamps	Channels	Sampling Frequency	# of Classes
**EEG**	26	9360	500	30	100 Hz	2
**fNIRS**	26	9360	25	72	5 Hz	2

**Table 2:** **Subject-Dependent Results.** Subject-dependent results for subjects 1, 2, and 3. This setting aims to determine whether the model can learn specific features from brain activities. The bold value stands for the best result among all models.

Modality	Models	Accuracy	Precision	Recall	F1	AUROC
**fNIRS**	**SVM**	92.44±8.71	92.99±8.98	91.97±8.42	92.26±8.98	92.56±8.51
	**RF**	92.44±2.83	91.76±5.20	93.75±0.45	92.57±2.93	92.65±2.74
	**KNN**	82.10±15.38	82.85±16.91	81.40±15.43	82.02±16.10	81.91±15.10
	**Resnet50**	88.43±13.57	89.41±12.43	86.51±16.79	87.88±14.70	89.42±14.62
	**VGG16**	92.90±7.53	93.28±7.65	92.44±7.95	92.75±7.87	95.82±4.95
	**EF-Net**	99.69±0.27	100.00±0.00	99.39±0.53	99.69±0.27	99.80±0.33
**EEG**	**SVM**	71.14±11.81	73.14±12.51	72.44±8.98	72.42±10.13	71.40±11.67
	**RF**	84.57±3.08	88.24±4.63	80.79±3.31	84.30±3.94	84.69±3.38
	**KNN**	75.15±8.86	77.88±4.88	71.22±21.35	73.31±13.91	74.93±8.32
	**Resnet50**	93.83±5.40	93.14±8.10	95.17±3.55	93.99±5.36	95.90±4.73
	**VGG19**	97.38±2.55	97.64±1.27	97.19±4.03	97.36±2.71	98.02±1.84
	**EF-Net**	96.45±2.38	97.28±1.85	95.89±3.29	96.53±2.26	97.09±1.70
**fNIRS+EEG**	**SVM**	86.27±6.11	87.25±4.50	85.74±8.43	86.30±6.69	86.19±5.70
	**RF**	86.88±8.16	90.47±5.95	82.83±12.22	85.91±9.99	87.11±7.80
	**KNN**	74.69±8.80	78.38±4.47	68.92±20.99	72.24±13.97	74.43±8.21
	**V16 + R50**	94.60±6.95	94.47±8.00	94.91±5.69	94.57±7.02	96.44±5.13
	**V16 + V19**	97.53±0.71	97.75±2.03	97.49±3.62	97.53±0.90	98.30±0.98
	**EF-Net**	99.38±0.71	99.72±0.49	99.03±0.98	99.36±0.73	99.70±0.41

**Table 3:** **Subject-Semidependent Results.** Samples from all subjects (1–26) were shuffled into an 80–20 training–testing split. This setting assesses the model’s ability to generalize to different subjects. The bold value stands for the best result among all models.

Modality	Models	Accuracy	Precision	Recall	F1	AUROC
**fNIRS**	**SVM**	77.69±1.05	77.11±1.15	79.15±1.51	78.12±1.27	77.67±1.04
	**RF**	88.25±0.69	87.53±0.40	89.39±0.84	88.45±0.59	88.24±0.71
	**KNN**	80.45±1.66	78.58±1.99	84.08±1.27	81.24±1.63	80.43±1.63
	**Resnet50**	95.43±0.73	95.76±0.73	95.11±0.80	95.44±0.76	97.90±0.19
	**VGG16**	95.82±0.22	95.22±0.26	96.53±0.35	95.87±0.25	98.01±0.34
	**EF-Net**	98.48±0.29	98.31±0.16	98.69±0.69	98.50±0.30	99.55±0.14
**EEG**	**SVM**	67.88±1.51	69.50±2.36	64.51±0.91	66.91±1.58	67.90±1.55
	**RF**	69.84±1.11	70.02±1.09	70.07±1.51	70.04±1.28	69.83±1.11
	**KNN**	64.92±0.92	69.99±2.74	53.24±1.07	60.45±0.49	65.02±1.03
	**Resnet50**	87.29±1.02	87.30±0.25	87.46±2.71	87.36±1.34	93.42±0.49
	**VGG19**	92.96±0.47	92.31±0.40	93.84±1.41	93.06±0.57	96.61±1.16
	**EF-Net**	92.66±1.36	91.79±2.32	93.85±0.96	92.79±1.33	96.71±0.80
**fNIRS+EEG**	**SVM**	73.99±1.64	74.39±2.25	73.71±0.96	74.05±1.60	73.99±1.64
	**RF**	80.29±1.44	79.65±2.06	81.75±1.42	80.68±1.41	80.29±1.43
	**KNN**	64.92±0.92	69.99±2.74	53.24±1.07	60.45±0.49	65.02±1.03
	**V16 + R50**	93.79±3.98	94.82±1.92	92.59±6.61	93.65±4.32	96.59±2.50
	**V16 + V19**	93.91±1.11	93.77±1.27	94.17±0.89	93.97±1.07	95.98±1.37
	**EF-Net**	98.29±0.24	97.93±0.04	98.70±0.42	98.31±0.22	99.46±0.21

**Table 4:** **Subject-Independent Results.** A total of 26 Subjects were partitioned into twenty used exclusively for the training set and six used exclusively for the testing set. This setting aims to outline the model’s performance on unseen subjects. The bold value stands for the best result among all models.

Modality	Models	Accuracy	Precision	Recall	F1	AUROC
**fNIRS**	**SVM**	61.94±2.36	63.10±2.71	57.59±2.45	60.21±2.44	61.94±2.36
	**RF**	58.80±2.46	59.53±2.55	54.85±3.24	57.09±2.87	58.80±2.46
	**KNN**	53.73±1.73	53.58±1.69	56.36±1.35	54.92±1.27	53.73±1.73
	**Resnet50**	59.23±0.40	60.54±1.03	53.15±2.06	56.58±0.76	61.69±0.80
	**VGG16**	57.27±0.09	56.03±0.64	68.05±7.18	61.32±2.57	57.74±0.74
	**EF-Net**	62.78±1.05	62.13±1.43	65.65±2.69	63.80±1.10	65.48±1.53
**EEG**	**SVM**	59.38±0.51	61.85±0.96	49.10±3.19	54.69±1.73	59.38±0.51
	**RF**	57.39±1.44	57.89±1.80	54.44±1.79	56.10±1.23	57.39±1.44
	**KNN**	52.58±0.67	53.95±0.94	35.06±2.22	42.48±1.85	52.58±0.67
	**Resnet50**	56.47±0.33	59.26±1.30	49.04±8.93	55.63±3.85	58.79±0.39
	**VGG19**	59.89±1.69	61.05±1.99	53.83±3.50	57.91±2.27	61.44±1.07
	**EF-Net**	60.80±1.21	63.32±1.23	51.36±3.90	56.66±2.51	64.71±0.38
**fNIRS + EEG**	**SVM**	62.84±1.05	66.20±1.30	52.47±1.28	58.54±1.25	62.84±1.05
	**RF**	59.10±0.37	60.02±0.40	54.54±0.93	57.15±0.58	59.10±0.37
	**KNN**	52.58±0.67	53.95±0.94	35.06±2.22	42.48±1.85	52.58±0.67
	**V16 + R50**	60.76±0.89	60.56±1.00	61.85±4.96	61.12±2.28	63.63±0.70
	**V16 + V19**	60.14±1.50	58.97±2.35	67.87±6.42	62.92±1.88	62.46±2.33
	**EF-Net**	64.65±2.11	64.29±1.93	65.84±3.01	** 65.05±2.37 **	67.81±2.75

## Data Availability

We release the source code for data analysis at https://github.com/DL4mHealth/EF-Net. Please refer to [17] for the EEG-fNIRS dataset.

## Appendix A

**Table A1:** **Deep Learning Parameters.** The parameters selected for the deep learning baselines and our EF-Net when utilizing multimodal fNIRS and EEG data. Comput Time stands for computation time per epoch; SD, SSD, and SI stand for subject-dependent, subject-semidependent, and subject-independent, respectively.

Modality	Models	Settings	# of Epochs	Learning Rate	Comput Time	Batch Size
**fNIRS**	**Resnet50**	**SD**	60	$1\times 10^{-3}$	1 s	32
		**SSD**	100	$1\times 10^{-3}$	8 s	64
		**SI**	40	$5\times 10^{-5}$	7 s	64
	**VGG16**	**SD**	60	$1\times 10^{-5}$	270 ms	32
		**SSD**	100	$1\times 10^{-4}$	3 s	64
		**SI**	40	$5\times 10^{-5}$	3 s	64
	**EF-Net**	**SD**	60	$1\times 10^{-2}$	270 ms	32
		**SSD**	70	$1\times 10^{-3}$	1 s	64
		**SI**	40	$5\times 10^{-5}$	1 s	64
**EEG**	**Resnet50**	**SD**	100	$1\times 10^{-4}$	2 s	32
		**SSD**	60	$5\times 10^{-5}$	10 s	64
		**SI**	50	$5\times 10^{-5}$	9 s	64
	**VGG19**	**SD**	100	$1\times 10^{-4}$	405 ms	32
		**SSD**	60	$5\times 10^{-5}$	10 s	64
		**SI**	50	$5\times 10^{-5}$	7 s	64
	**EF-Net**	**SD**	60	$1\times 10^{-3}$	297 ms	32
		**SSD**	60	$5\times 10^{-4}$	5 s	64
		**SI**	50	$1\times 10^{-4}$	5 s	64
**fNIRS + EEG**	**V16 + R50**	**SD**	70	$5\times 10^{-5}$	1 s	32
		**SSD**	100	$1\times 10^{-4}$	13 s	32
		**SI**	100	$1\times 10^{-5}$	30 s	6
	**V16 + V19**	**SD**	70	$5\times 10^{-5}$	1 s	32
		**SSD**	60	$1\times 10^{-4}$	10 s	32
		**SI**	100	$1\times 10^{-5}$	17 s	64
	**EF-Net**	**SD**	70	$1\times 10^{-3}$	315 ms	32
		**SSD**	60	$1\times 10^{-2}$	6 s	32
		**SI**	100	$5\times 10^{-5}$	5 s	64

**Table A2:** **Parameter Tuning for Subject-Dependent Experiments with EEG-fNIRS Modalities.** The three different parameters tuned for each ML baseline model.

Baseline Type	Parameter	Accuracy	Precision	Recall	F1	AUROC
**SVM**	**C = 0.5**	75.77±7.39	76.54±8.78	78.04±3.02	76.97±6.15	75.70±7.14
	**C = 1.0**	86.27±6.11	87.25±4.50	85.74±8.43	86.30±6.69	86.19±5.70
	**C = 1.5**	88.78±3.54	91.08±4.73	89.68±6.33	90.30±5.54	90.26±4.93
**RF**	**Trees = 50**	88.73±4.64	89.90±4.42	87.44±5.86	88.59±5.10	87.84±5.45
	**Trees = 100**	86.88±8.16	90.47±5.95	82.83±12.22	85.91±9.99	87.11±7.80
	**Trees = 200**	91.51±2.55	93.42±1.40	89.50±4.64	91.33±3.08	91.39±2.45
**KNN**	**K = 5**	74.69±8.80	78.38±4.47	68.92±20.99	72.24±13.97	74.43±8.21
	**K = 10**	66.51±4.20	67.91±0.93	67.80±14.96	67.05±7.62	67.31±6.67
	**K = 13**	65.74±4.42	70.14±0.43	58.81±14.36	63.51±9.48	65.78±4.20

**Table A3:** **Parameter Tuning for Subject-Semidependent Experiments with EEG-fNIRS Modalities.** The three different parameters tuned for each ML baseline model.

Baseline Type	Parameter	Accuracy	Precision	Recall	F1	AUROC
	**C = 0.5**	70.71±1.34	71.88±2.21	68.73±0.55	70.26±1.32	70.73±1.37
**SVM**	**C = 1.0**	73.99±1.64	74.39±2.25	73.71±0.96	74.05±1.60	73.99±1.64
	**C = 1.5**	75.82±1.38	75.91±1.98	76.15±0.77	76.03±1.28	75.82±1.39
	**Trees = 50**	78.22±1.55	76.63±2.00	81.64±1.01	79.05±1.51	78.20±1.53
**RF**	**Trees = 100**	80.29±1.44	79.65±2.06	81.75±1.42	80.68±1.41	80.29±1.43
	**Trees = 200**	82.10±1.76	82.30±1.85	82.10±1.94	82.20±1.85	82.11±1.76
	**K = 5**	64.92±0.92	69.99±2.74	53.24±1.07	60.45±0.49	65.02±1.03
**KNN**	**K = 10**	62.36±0.54	65.17±2.01	54.38±1.75	59.25±0.18	62.43±0.62
	**K = 13**	61.63±0.83	69.25±2.69	43.00±3.10	52.97±1.93	61.78±0.86

**Table A4:** **Parameter Tuning for Subject-Independent Experiments with EEG-fNIRS Modalities.** The three different parameters tuned for each ML baseline model.

Baseline Type	Parameter	Accuracy	Precision	Recall	F1	AUROC
	**C = 0.5**	62.82±0.81	66.44±1.72	51.98±2.41	58.28±1.21	62.36±1.34
**SVM**	**C = 1.0**	62.84±1.05	66.20±1.30	52.47±1.28	58.54±1.25	62.84±1.05
	**C = 1.5**	62.28±1.08	65.37±1.23	52.22±1.40	58.06±1.35	62.28±1.08
	**Trees = 50**	57.96±1.00	58.23±0.93	56.30±1.70	57.24±1.30	57.96±1.00
**RF**	**Trees = 100**	59.10±0.37	60.02±0.40	54.54±0.93	57.15±0.58	59.10±0.37
	**Trees = 200**	59.95±0.41	61.26±0.64	54.17±0.24	57.49±0.14	59.95±0.41
	**K = 5**	52.58±0.67	53.95±0.94	35.06±2.22	42.48±1.85	52.58±0.67
**KNN**	**K = 10**	53.89±2.10	55.27±2.74	40.52±3.15	46.75±3.04	53.89±2.10
	**K = 13**	53.98±2.34	56.89±3.76	32.47±3.59	41.33±3.88	53.98±2.34
